# Enhanced Aromatic Profile and Functionality of Cheese Whey Beverages by Incorporation of Probiotic Cells Immobilized on *Pistacia terebinthus* Resin

**DOI:** 10.3390/foods9010013

**Published:** 2019-12-22

**Authors:** Vasiliki Schoina, Antonia Terpou, Aikaterini Papadaki, Loulouda Bosnea, Nikolaos Kopsahelis, Maria Kanellaki

**Affiliations:** 1 Food Biotechnology Group, Department of Chemistry, University of Patras, GR-26500 Patras, Greece; schoina_vasiliki@hotmail.com (V.S.); m.kanellaki@upatras.gr (M.K.); 2 Department of Food Science and Technology, Ionian University, 28100 Kefalonia, Greece; kpapadaki@aua.gr (A.P.); kopsahelis@upatras.gr (N.K.); 3 Hellenic Agricultural Organization DEMETER, Dairy Research Institute, 45221 Ioannina, Greece

**Keywords:** functional whey beverage, cheese whey, *Pistacia terebinthus* resin, probiotics, immobilization, terpenes

## Abstract

In the present study, cheese whey was utilized for the development of a novel functional beverage, using *Lactobacillus casei* ATCC 393 probiotic cells immobilized on *Pistacia terebinthus* resin (pissa Paphos). Evaluation of shelf life of the produced beverages showed that spoilage microorganisms were not observed in beverages containing *P. terebinthus* resin. Terpenes’ rich content might have contributed to the antimicrobial activity of the produced beverages; however, no significant effect on the viability of the immobilized probiotic cells was obtained. Whey beverages containing the immobilized biocatalyst retained a high viability (>1 × 10^6^ CFU/g) of probiotic cells during a storage period of 30 days at 4 °C. The superiority of whey beverages containing the immobilized biocatalyst was also highlighted by GC-MS analysis, while the enhanced aromatic profile, which was mostly attributed to the higher concentration of terpenes, was also detected during the sensory evaluation performed. Conclusively, this study indicated the high commercialization potential of these novel functional whey beverages, within the frame of a sustainable dairy waste valorization approach. To the best of our knowledge, this is the first food-oriented approach within the guidelines of the circular economy reported in the literature, using the autochthonous *Pistacia terebinthus* resin for the production of functional whey beverages.

## 1. Introduction

Cheese whey is the main liquid by-product of the dairy industry, which contains many valuable constituents; while its approximate amount during cheese production may range between 8 and 9 L per kg of produced cheese, depending on cheese yield or type of processed milk [1,2]., Cheese whey may be discarded as a waste to the environment, representing an important polluting problem due to its high organic load [3]. The applications of cheese whey valorization are rather limited; concerning the industrial relevance, only a small portion of whey by-product is used for animal feed or food applications, while the rest is discarded to the environment [4]. The disposal of these effluents without an appropriate pretreatment can pose serious environmental hazards [2]. On the other hand, recent reports have revealed the importance of cheese whey composition and proposed alternative valorization options, aiming to establish a sound basis and take advantage of its high nutritional and functional value content [1,5]. Cheese whey retains approximately 50%–55% of milk nutrients, and thus it can be used as a potential resource for various value-added products [4]. More specifically, cheese whey could be a valuable food itself, as it is a source of high-quality proteins, minerals, vitamins, and lactose [5,6]; however, cheese whey is not preferred by consumers in its current form, which is mostly due to its unpleasant flavor. The latest research efforts target its transformation into a wide range of valuable products through the application of different technological processes [7]. Specifically, the valorization of cheese whey has been proposed as a substrate for food production, such as whey cheeses and whey-based beverages, or as a potential feedstock for the production of valuable compounds through fermentation processes, such as probiotic starter cultures, baker’s yeast, single cell protein, and biofuel production [5].

More specifically, a constantly increasing interest has been noted lately, regarding whey/dairy beverages and consumers’ preference [8,9,10]. Whey-based beverages are an excellent way to reuse the liquid whey produced from cheese manufacturing [5]. The term ‘whey beverage’ has been used to describe a drinkable product based on liquid cheese whey by-product as the main or as the most significant component [11]. Non-alcoholic whey beverages include a wide range of products manufactured by mixing native sweet, acid whey, or diluted whey with different additives such as fruits, fruit juices, crops and crop by-products, prebiotics, chocolate, and other aromatic agents [11,12,13,14,15]. This kind of ready-to-drink beverage is very common and popular among consumers and can lead to profitable and sustainable processes, especially when incorporated together with other functional ingredients [5,15].

Approaches that can increase the resistance and viability of sensitive probiotic bacteria against adverse conditions have been already proposed, including the appropriate selection of acid and bile resistant strains, the addition of prebiotics, stress adaptation, cell immobilization, and drying (freeze-drying, spray-drying) [16,17]. Among them, immobilization has been previously reported as a technology that can provide protection to these sensitive cultures throughout manufacture, storage, freezing conditions, and during transit through the gastrointestinal tract [18,19,20].

In previous studies, resins from different tree species have been successfully employed for the encapsulation of lactobacilli and yeast cells [21,22,23,24,25]. The most popular resins originate from Pistacia plants (*Pistacia lentiscus, Pistacia terebinthus*), which are mostly native to the Mediterranean region [26]. The immobilization of probiotics on resins provides protection to the bacterial cells, especially when they are exposed to stressful environmental conditions such as low pH, gastrointestinal conditions, high salinity, and heat treatment [21,22,23]. According to a recent study, the resin deriving from *Pistacia lentiscus* was used as a microencapsulating and matrix-forming material for sustained drug release [27]. In addition, a more recent study revealed that *Pistacia lentiscus* mastic extract shows no toxic effects in Caco-2 cells, while in parallel, it may improve the intestinal barrier function [28]. Caco-2 cells are widely used to study human absorption and transport processes, as they are human adenocarcinoma cells. Likewise, mastic gum has been lately assigned as a traditional herbal medicine for oral and cutaneous use for the treatment of mild dyspeptic disorders, minor inflammations of the skin, and as an aid in healing of minor wounds, by the European Medicines Agency (EMA) [29], whereas several studies reported that it is safe and well-tolerated by humans, contributing to the smooth operation of the gastrointestinal system [27,30,31]. Moreover, other related species such as *P. atlantica*, *P. palaestina*, and *P. terebinthus* produce a resin similar to mastic [32]. All these resins have been widely studied for their health effects in several food applications, such as bakery products, chewing gums, liquor, flavored wines, flavored water, and filter coffee [32]. Resins in general are suitable matrix materials for probiotic bacteria immobilization, providing in parallel health benefits to the consumers.

The resin deriving from *Pistacia terebinthus,* which is commonly known as pissa Paphos, is widely distributed in most coastal and inner regions of Cyprus. Pissa Paphos has a crystalline form and is secreted from the thorium tree trunk, where it appears as a tear in the engraved points of the tree. Pissa Paphos has recently been reported to contain specific components, such as terpenoids, which have been associated with anti-inflammatory and antimicrobial effects. Moreover, natural phenols and flavonoids have been also related to potential antioxidant and anticancer activities [33]. Still, Pissa Paphos has not been widely investigated as immobilization support.

The aim of the present work was to take advantage of the compositional and textural properties of Pissa Paphos for the production of a novel whey-based functional beverage. In the frame of that, *L. casei* ATCC 393 probiotic cells have been immobilized on Pissa Paphos, freeze-dried, and subsequently used in cheese whey fermentations. The obtained results pointed out the antimicrobial and biopreservative synergistic action of the immobilized biocatalyst, together with the enhanced aromatic character of the produced beverages. Likewise, the present study proposes an innovative and sustainable cheese whey valorization process toward the production of functional beverages.

## 2. Materials and Methods

### 2.1. Immobilized Probiotic Biocatalyst

The Gram-positive probiotic bacterial strain *Lactobacillus casei* ATCC 393 (DSMZ, Braunschweig, Germany) was selected for the production of the immobilized biocatalyst. *L. casei* biomass was grown under anaerobic conditions at 37 °C for 48 h in MRS broth (LabM, Heywood, UK). Wet biomass was harvested by centrifugation (Sigma 3K12, Bioblock Scientific, Osterode am Harz, Germany) at 5000 rpm for 10 min at 25 °C. The harvested biomass of *L. casei* ATCC 393 was introduced in MRS broth along with freeze-dried particles of *Pistacia terebinthus* resin (Pissa Paphos), which were priory sterilized and remained at 37 °C for 48 h [22]. When the medium was exhausted (sugar content <0.1%), the fermented liquid was decanted, and the probiotic biocatalyst was washed twice with sterile 1/4 strength Ringer’s solution (LabM) for the removal of free cells [22]. The probiotic immobilized biocatalyst was frozen without cryoprotectants at −45 °C (cooling rate 5 °C/min), and freeze-dried for 48–72 h at −45 °C and 5 × 10^−3^ mbar in a Freeze-Drying System, Freezone 4.5 (Labconco, Kansas City, MI, USA). All media were autoclaved at 120 °C at 1–1.5 atm for 15 min prior to use.

### 2.2. Functional Whey Beverages Production

Deproteinized cheese whey with the following composition (*w/v*): <0.1% fat, 0.4% total protein, 4.7% total carbohydrates, and pH 6.4, was supplied by a local cheese factory (A.VI.GAL SA-Achaia milk industry) as an industrial by-product of feta cheese production (after Myzithra cheese production) and used as a base material to produce functional whey beverages. Initially, cheese whey was diluted with tap water (1:1, *v/v*), pasteurized at 65 °C for 30 min, and then remained at room temperature to cool down. Whey was filtered, and its pH value was adjusted to 3.9 by the addition of 1 g/L citric acid (Merck, Taufkirchen, Germany), which is a food-grade material used as an aromatic and preservative in foods and non-alcoholic beverages [34]. Subsequently, cheese whey was placed into sterile glass containers (a total volume 200 mL), and the probiotic biocatalyst was incorporated at various concentrations (A1: 0.3; A2: 0.6; A3: 1.2 g per 100 mL of whey beverage) using continuous stirring. For comparison reasons, whey beverages were also prepared only with the addition of *Pistacia terebinthus* resin (Pissa Paphos) using the following concentrations: B1: 0.3; B2: 0.6; and B3: 1.2 g per 100 mL of whey beverage. Finally, deproteinized cheese whey was used as a control sample without any further treatment (sample C). All products were stored at 4 °C for 30 days.

### 2.3. Whey Beverages Microbiological Profile

Samples of 10 g each of sour milk were diluted into 100 mL of sterile 1/4 strength Ringer’s solution (LabM) and mixed in a stomacher (Bagmixer 400, Model VW, Interscience, Saint Nom, France). Subsequently, the appropriate serial dilutions were prepared by sterile Ringer’s solution ¼ strength. Viable counts for yeasts and fungi, coliforms, enterobacteria, Salmonella, and staphylococci were performed in triplicate by pour plating 0.1 mL or 1 mL of appropriate dilutions on the suitable (selective) media for each species and according to instructions given by the manufacturer [35]. Specifically, yeasts and fungi were determined by plating on potato dextrose agar (PDA) (Fluka, Buchs, Switzerland) after incubation at 30 °C for 72 h. Coliforms were enumerated on violet red bile agar (LabM) after incubation at 30 °C for 24 h. Total enterobacteria were enumerated on violet red bile glucose agar (VRBGA) (LabM) after incubation at 37 °C for 24 h. Staphylococci counts were performed on Baird–Parker agar with added egg yolk tellurite medium (BP) (LabM) after incubation at 37 °C for 48 h, while possible counts of *Staphylococcus aureus* could be identified by plate counting on the first 24 h of incubation. Viable cells of *L. casei* were enumerated on MRS-V agar containing 1% vancomycin antibiotic (Fluka) and expressed as the percentage survival of probiotic cells during 30 days of storage (4 °C) [19].

### 2.4. Physicochemical Analysis

The pH values of whey beverages were measured using a digital pH meter by direct immersion of the electrode (ΕPI-BION SENTRON pH-System 1001, Kamerlingh-Onnesstraat, Netherlands). Titratable acidity was determined using 0.1 mol L^−1^ NaOH (Sigma-Aldrich Ltd., St Louis, MI, USA), and phenolphthalein was used as an indicator and expressed as g of lactic acid per 100 mL of whey beverage.

Sugars (glucose, galactose, and lactose) were determined by high-performance liquid chromatography (HPLC). All samples were diluted at 1% (*v/v*) and filtered through a 0.2 nm disposable cellulose acetate filters (Chromafil), and 40 μL of the filtrates were injected directly into the column. A Shimadzu HPLC system (Kyoto, Japan) equipped with a SCR-101N stainless steel column, a LC-9A pump, a CTO-10A oven at 60 °C, and a RID-6A refractive index detector was used for the analysis. Ultra-pure water obtained by a Milli-Q water purifier system (resistivity 18.2 MΩ cm^−1^) with a flow rate of 0.8 mL min^−1^ was used as a mobile phase, and 1-butanol (0.1%, *v/v*) (Sigma-Aldrich Ltd.) was used as an internal standard. Sugar concentrations were calculated using standard curves.

### 2.5. Analysis of Aroma Volatiles by Solid-Phase Microextraction Gas Chromatography–Mass Spectrometry

Selected whey beverages were analyzed after 30 days of storage (at 4 °C) and compared with control samples (C). For solid-phase microextraction gas chromatography/mass spectrometry (SPME GC/MS) analysis, 7 mL of beverage samples (B2, C) were collected and mixed with 3 g of salt (Sigma-Aldrich Ltd., St Louis, USA) [36]. The samples were placed into a 20-mL headspace vial, which was sealed with a rubber septum and heated in a water bath for approximately 5 min for the temperature to be stabilized at 60 °C. Then, each sample was heated (60 °C) for 45 min, while the SPME needle (bearing a 2-cm fiber, 50/30 mm DVD/Carboxen/PDMS Stable Flex Supelco, Bellefonte, state of Pennsylvania, USA) was inserted through the septum, and the fiber was exposed to the headspace. The volatiles absorbed from the SPME needle were analyzed by GC/MS (Shimadzu GC-17A, MS QP5050; Shimadzu, Kyoto, Japan with a capillary column Supelco CO Wax-10; 60 m, 0.32 mm i.d., 0.25-μm film thickness; Merck, Darmstadt, Germany) coupled with a GCMS-QP5050A mass spectrometer. Helium was used as the carrier gas (linear velocity of 1.5 mL min^−1^). The oven temperature was programmed at 35 °C for 3 min, 5 °C min^−1^ to 110 °C, and then 10 °C min^−1^ to 240 °C, after which it was retained at 240 °C for 10 min. The injector operated in a spitless mode. The injector and detector temperatures were programmed at 280 °C and 250 °C, respectively. The mass spectrometer was operated in electron impact mode with the electron energy set at 70 eV.

The identification of aromatic compounds was achieved by comparison with standard compounds and data obtained from NIST107, NIST21 (www.nist.gov), and SZTERP (Shimadzu Instruction Manual) libraries. The results were expressed as the percentage of the total identified compounds [22].

### 2.6. Sensory Evaluation

Sensory evaluation was carried out by a group of 10 adult laboratory members (five men and five women) aged between 22 and 55. Whey beverages (C, A2, B2) were assessed for their sensory characteristics regarding appearance, flavor, texture, acidity, sweetness, mastic odor, cheese odor, and overall acceptability. The intensity of the studied attributes was conducted on a 0–10 cm unstructured linear scale (the higher the number, the greater the intensity) anchored with the words “high intensity” and “absence” on the right and left ends, respectively [22]. Whey beverages were evaluated on the 30th day of storage at 4 °C. Evaluations were conducted at ambient room temperature under standard fluorescent light. The samples were placed into glass containers of equivalent amounts (50 mL), coded randomly by three-digital numbers, and served cool (~4 °C). Evaluators used bread and low mineral content water to neutralize and clean their mouths between sample testing. The results are presented as a star chart of the product’s attributes.

### 2.7. Experimental Design and Statistical Analysis

All the experiments were performed in duplicate, all analyses were carried out in triplicate, and the results are presented as mean values ± standard deviation. The statistical differences among treatments were estimated by analysis of variance (ANOVA). Whenever ANOVA indicated a significant difference between variables at a significance level of 5% (*p* < 0.05), the Tukey’s HSD (honest significant difference) test was carried out using the Analysis ToolPak of Microsoft^®^ Excel^®^ 2016 software (Microsoft, Washington, DC, USA).

## 3. Results and Discussion

### 3.1. Microbiological Characteristics and Probiotics Viability

*L. casei* ATCC 393 is a probiotic bacterium that has been added in many dairy products such as cheeses [22,37], fermented beverages [38], frozen desserts [19], and yogurts [39]. The selected strain has been applied either as a starter or as an adjunct probiotic culture mainly due to its health potential and its stability during manufacture and storage as well as due to its high survival rates reported under simulated gastrointestinal conditions [19,36]. In vitro and preliminary in vivo studies have demonstrated that *L. casei* ATCC 393 displayed several probiotic properties such as cholesterol removal in parallel with pathogens reduction and more importantly distinct adhesion in rat intestinal mucosa [40,41].

**Figure 1:**
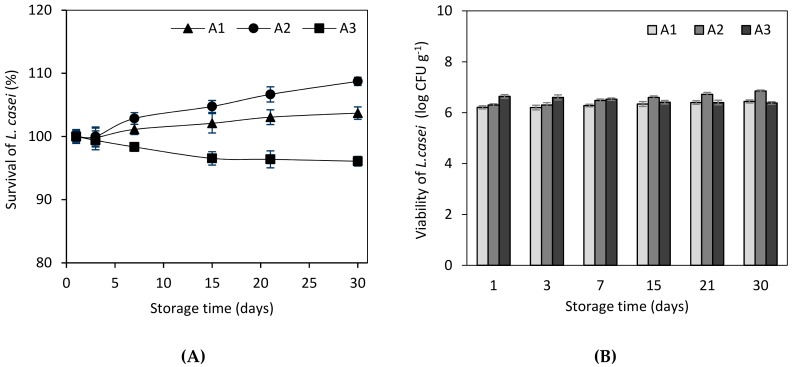
Viability of the immobilized probiotic cells (*L. casei* ATCC393 immobilized on *Pistacia terebinthus* resin) added at various concentrations (A1: 0.3 g, A2: 0.6 g, and A3: 1.2 g per 100 mL of whey) during whey beverages cold storage (4 °C) for 30 days (viability expressed as (**A**) % survival and (**B**) log cfu gr^−1^).

As shown in Figure 1, the survival of *L. casei* reduced by 4% during 30 days of storage in the case of sample A3. This outcome may come as a result of the higher amount of incorporated resin (1.2 g per 100 mL whey beverage) in sample A3, providing an increased terpene concentration, which may have acted against probiotic cells. On the other hand, the viability of probiotic cells increased during storage in both samples A1 and A2 (Figure 1). Specifically, samples A1 and A2 showed a 3.7% and 8.7% increase of cell viability, respectively, by the 30th storage day. Likewise, the obtained results showed that the survival of *L. casei* might be promoted by using lower concentrations of the resin. Nevertheless, a sharp decrease was observed in the case of 1.2 g of resin per 100 mL of whey; thus, the survival of *L. casei* cells seems to be negatively affected after a critical *P. terebinthus* concentration. High survival rates of probiotic cells (>6 log cfu mL^−1^) are mandatory in food products targeting to provide their beneficial effects to the consumer [20]. In addition, a more recent study showed that probiotic whey beverages may offer protection against *Salmonella typhimurium* infection in mice, verifying the importance of probiotic cells viability as a health factor [42]. In the present study, whey beverages enriched with 0.3 g (A1) and 0.6 g (A2) per 100 mL of whey were proved to be the most successful treatments regarding probiotic viability, while sample A2 showed the highest viability rates between all samples (Figure 1). In a similar approach, Ambrosio et al. highlighted that citrus terpenes showed great activity against pathogenic bacteria, without affecting beneficial bacteria [43]. As a result, citrus terpenes were reported to provide the best selective antimicrobial effect between pathogenic and beneficial bacteria [43]. Likewise, in the present study, *Pistacia terebinthus* resin along with its high terpene concentration provided an antimicrobial effect against possible pathogenic or spoilage microorganisms and had a minimum effect on the immobilized probiotic culture.

Samples of whey beverages were also tested for their microbial stability regarding yeast and fungi, staphylococci, enterobacteria, and coliforms as a shelf-life indicator, during a refrigerated storage period of 30 days. The control whey beverage presented significantly (*P* > 0.05) higher amounts of yeast and fungi (1.03 log cfu g^−1^) from the first storage day, which increased significantly (*P* > 0.05) at the 30th storage day (4.16 log cfu g^−1^). The accumulation of yeast and fungi in pasteurized whey may come as a result of air or facilities contamination, as they are the most frequent contaminant of food products [44]. On the contrary, no spoilage or possible pathogenic microorganisms were detected during the storage of whey beverages with the incorporated resin, either added alone (B1, B2, B3) or as an immobilized biocatalyst (A1, A2, A3). These results are in accordance with previous studies indicating that mastic resins may provide antimicrobial characteristics when incorporated within dairy products as a result of their terpene abundance [22,23]. Regarding this subject, it has been well documented that terpenes, terpenoids, and phenylpropenes are considered as the most active antimicrobial compounds when incorporated in dairy products [23,45,46].

### 3.2. Physicochemical Characteristics of Whey Beverages

All whey beverages were evaluated concerning their lactose, glucose, and galactose concentration, pH value, and total acidity, and the results are presented in Table 1. A slight increase in total acidity and a parallel pH decrease in whey beverages were observed in the case of the incorporated probiotic biocatalyst (A1, A2, and A3) as compared to whey beverages with only resin (B1, B2, and B3), which showed a more likely constant total acidity and pH value. Lactose content was slightly decreased during the storage of whey beverages containing the immobilized biocatalyst (A1 A2, and A3) due to the viable probiotic cells, which are known to consume lactose even at harsh conditions (low temperature, low pH) producing lactic acid [47]. Likewise, lactose was hydrolyzed to glucose and galactose only in the case of whey beverages containing the immobilized biocatalyst. Glucose could be characterized as a preferred carbon source compared to galactose; hence, a significant decrease in glucose content was obtained during the 30 days of storage, while the amounts of galactose were higher in all cases. Glucose is an easily metabolized sugar and is mostly preferred by microorganisms as energy source [39]. Nevertheless, a slight reduction of glucose has been also observed in control beverages at the 30th day of storage, which may be explained due to yeast and fungi accumulation, as reported in Section 2.2.

### 3.3. Effect of Pistacia Terebinthus Resin on Volatile By-Products of Functional Whey Beverages

Terpene hydrocarbons can serve as effective flavoring ingredients providing citrus, pine, balsamic, woody, and fruity notes when detected in food matrices [48]. As it has been well documented, terpenes deriving from essential oils can serve as natural antimicrobial agents [45,49] as well as effective flavoring ingredients [48,50] when incorporated into food products. Whey beverages showing the optimum probiotic viability (A2) during storage (4 °C) were analyzed after the 30th day of production for their terpene content by SPME/GC-MS technique and compared with the respective whey beverages containing only resin (B2) and control whey beverages (C).

**Figure 2:**
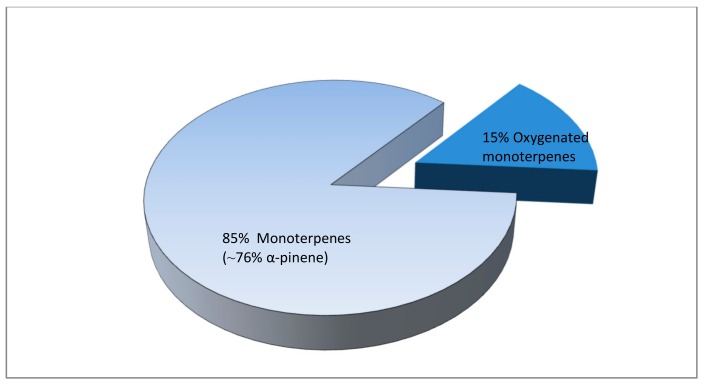
Diagram of terpene hydrocarbons of whey beverage (A2) presented as the percentage of each category.

As expected, no terpenoid compounds were detected in control whey beverages (only traces of D-limonene and a-pinene), whereas in whey beverages containing the immobilized biocatalyst (A2) or the resin alone (B2) the analysis revealed eight and nine different monoterpenes and 10 oxygenated monoterpenes, respectively (Table 2). These results indicated that terpenoid compounds detected in whey beverages were derived from *Pistacia terebinthus* resin. Specifically, monoterpenes represented ~85% of the total terpenes, while oxygenated monoterpenes represented ~15% of the total terpenes in both cases (Figure 2). The most abundant representative of monoterpenes, as depicted in Table 2, was α-pinene (76.5% and 73.9% of total terpenoids) followed by β-pinene (3.6% and 4.2% of total terpenoids) and D-limonene (1.8% and 2.1% of total terpenoids) for the case of A2 and B2, respectively; this result was mostly expected, since these compounds are the most abundant in the essential oils of Pistacia tree species [23,51].

Most of the detected terpenes are characterized by exceptional aromatic characteristics and can contribute to the aroma and flavor of the produced beverages by neutralizing any negative aromatic characteristics of whey caused by the occurrence of phenolic compounds or short-chain fatty acids [52,53]. Especially, monoterpenes can provide unique floral and fruity aromatic characteristics to food products due to their low threshold values [22]. For example, α-pinene (75.6% of total terpenoids) is an important bicyclic terpenoid that is widely used in the fragrance industry and for the synthesis of a variety of chemicals, while it is mostly known for its pine odor [54]. D-limonene (1.8% of total terpenoids) is reported to be responsible for a citrus mint and could be derived not only from the resin but also from whey fermentation via the probiotic culture [55]. A similar aromatic effect can be provided via other monoterpenes also; for example, linalool (0.8% of total terpenoids) is known for its sweet floral orange odor, while 3-carene (0.7% of total terpenoids) is known for its sweet lemon odor [53,56].

More importantly, apart from their pleasant odor, the plethora of monoterpenes as well as some oxygenated monoterpenes has been reported to show multiple biological activities against various pathogens and provide beneficial health effects to the consumer [57]. Specifically, α-pinene and β-pinene, which were the most abundant monoterpenes detected in whey beverages, show high antimicrobial and antioxidant effects and could provide an important health impact. For instance, according to a recent study, *Pistacia atlantica* essential oil provided a protective effect against ethanol-induced gastric ulcer and an antibacterial effect against *Helicobacter pylori* with α-pinene being most likely the responsible agent of this biological activity [58]. Likewise, another study demonstrated that α-pinene may pose a significant anti-Leishmania activity [59]. Additionally, camphene (1.2% of total terpenoids), which is a bicyclic monoterpene, is considered a key compound, since it can provide various biological benefits such as anticancer, anti-inflammatory, antifungal and anti-gastric ulcer activity [60,61]. Among oxygenated monoterpenes, α-terpineol (4.8% of total terpenoids) was the most abundant, which presents an important antimicrobial effect against *E. coli* and *S. aureus* [46].

To conclude, terpenoid compounds are proposed to be considered not only as aromatic agents but more importantly as potential eco-friendly alternatives in food preservation targeting to prolong the shelf life of food products along with providing their beneficial health effects. In addition, the recent related studies dealing with efficient probiotic delivery [62], antioxidant activity [63], low-cost approaches to optimize sensory attributes [64], the evaluation of acceptance [65], and nutritional quality [66] point out the high commercialization potential of whey-based functional beverages.

Other than terpenoid compounds, 44 whey-derived volatile compounds were detected: 31 compounds in beverage C, 33 compounds in beverage B2, and 42 compounds in beverage A2 (Table 2). These compounds included 12 esters, 7 acids, 10 alcohols, 10 aldehydes, and 5 ketones.

The majority of the identified compounds were acids, in all cases. The organic acids detected were as follows: butanoic, hexanoic, 2-methyl-butanoic, 3-methyl-butanoic, octanoic, nonanoic, and decanoic acid. Organic acids are important flavor compounds, with a relatively high threshold value that mainly derive by the lipolysis of milk lipids [72]. Nonanoic and decanoic acids have been previously reported in skim milk powder as responsible compounds for the sweet, fatty, and buttery-like odors, while octanoic acid has also been previously reported in milk whey concentrate [73]. Likewise, 3 methyl-butanoic acid is characterized for its “sweet–cheesy” flavor. As one can observe, the majority of organic acids were detected in A2 whey beverage, a result mostly expected as this beverage was the one containing the immobilized biocatalyst.

Aliphatic primary alcohols such as hexanol may impact a fruity, nutty note to the flavor, while branched-chain primary alcohols indicated the reduction of aldehydes, (e.g., 3-methyl-1-butanol derives from reduction of the aldehyde produced by leucine). Secondary alcohols, such as 2-pentanol and 2-heptanol, are formed by enzymatic reduction of the corresponding methyl ketones, which themselves are derived from fatty acids by oxidation [68]. 1-Octen-3-ol, which has been detected in all samples, is known for its distinctive mushroom flavor and has been detected in ultrahigh temperature sterile milk and in oxidized dairy products [73]. Phenylethanol detected in all cases belongs to the most odorous aromatic alcohols, presenting rose flower notes, and may derive from phenylalanine [72]. Nevertheless, in all cases, hexanol was the main detected alcohol, which is in agreement with previous similar studies [74].

Aldehydes are also an important group of volatile compounds responsible for the formation of the characteristic aroma profile of dairy products [75]. Aldehydes are major oxidation products of unsaturated fatty acids. Among them, hexanal (found in all samples) is considered a typical marker of the oxidative processes and is characterized by an intrinsic leafy green smell [76], while most aldehydes are considered contributors of the metallic flavor attributes of liquid whey [71]. 2-octenal, which was found in all samples, is characterized by a cucumber flavor, while 3-methyl-butanal (also identified in all samples) presents a cocoa malty flavor. Likewise, benzaldehyde, which was detected in all cases and is usually present in milk whey concentrate, presents bitter almond aromatic notes [68]. Still, the main aldehyde identified in all cases was hexanal, as also previously reported [74].

Regarding esters, ethyl butanoate, ethyl hexanoate, and ethyl octanoate have been detected in all samples. Their presence, even in low concentrations, has been associated with fruity and floral aromas and flavors, and esters are considered important compounds due to their ability to reduce the effect of unpleasant odors that might be caused by phenolic compounds or short-chain fatty acids [69].

Μethyl-ketones have been previously reported as significant volatile organic compounds in dairy samples, with those between 5 and 13 carbon atoms (detected in all samples) contributing a flavor that resembles that of fruit [76]. For example, 2-heptanone contributes a green fruity flavor, while 2-nonanone contributes a malty fruity flavor. Methyl ketones are formed in a metabolic pathway during the fermentation with *Lactobacillus* strains, which are connected to the β-oxidation pathway [68]. 2,3-butanedione (diacetyl) is obtained from the condensation of two molecules of pyruvate during the valine and leucine biosynthesis pathways. Diacetyl is considered as a key odor compound for the buttery smell of the products. Its production is usually attributed to high production temperatures and/or the presence of various bacterial strains, while citrate is reported as a common substrate for its production [76]. It is worth noting that in our case, both high temperatures and citric acid are applied. Whey was obtained after myzithra type manufacture (which implies high production temperatures), while the initial pH of the beverages has been adjusted using citric acid. Hence, those facts justify the detection of diacetyl in the respective whey beverage samples.

### 3.4. Effect of Pistacia terebinthus Resin on Sensory Characteristics of Whey Beverages

**Figure 3:**
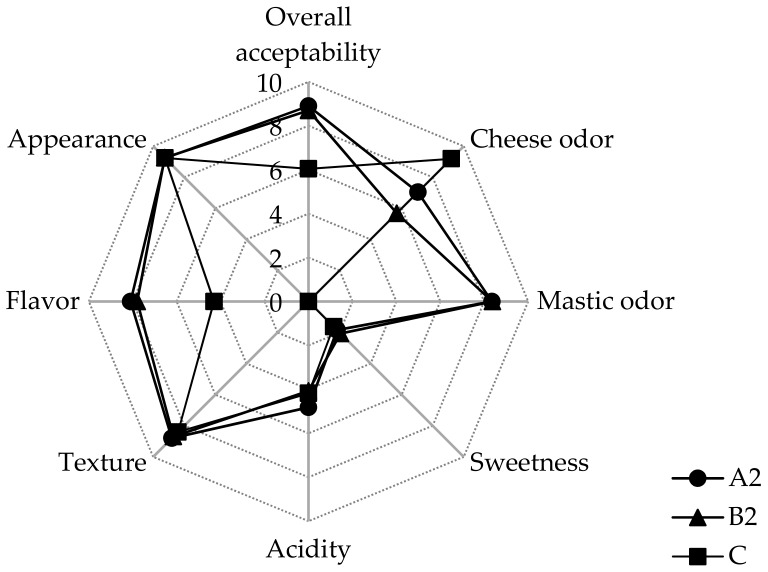
Sensory evaluation of whey beverages containing an immobilized biocatalyst on *P. terebinthus* resins (A2), *P. terebinthus* resins (B2), and control whey beverages at the 30th day of storage (4 °C).

Whey beverages (C, B2, A2) were assessed for their sensory characteristics on a 0–10 preference scale, and the results are illustrated in Figure 3.

As one can observe, it appears that the incorporation of the resin (Pissa Paphos) either single (B2) or an immobilized biocatalyst (A2) in whey beverages affected the preference of evaluators. Beverage samples containing the resin (B2, A2) were characterized by an enhanced sweet mastic odor. At this point, it should be highlighted that evaluators could detect the taste and the aromatic character of mastic in the beverages with the incorporated resin (B2, A2), but they could not feel any difference in the texture of the samples. This is a very important outcome as the freeze-dried particles of the resin seemed to be homogeneously dissolved within the beverage without causing any product dysfunction. In fact, all whey beverages with the incorporated resin were characterized as cool beverages with a pleasant characteristic mastic odor. Likewise, all evaluators reported a significant flavor improvement of the beverages containing the resin (B2, A2) compared to the control sample (C). This result brings practical information to the industrial area, highlighting the valorization potential of cheese whey by-products by the incorporation or aromatic resins.

## 4. Conclusions

*Pistacia terebinthus* resin demonstrates a great potential, as an ingredient or support, in the development of novel nutraceutical and functional products. This study presented the development of a novel functional whey beverage, with probiotic and antimicrobial characteristics, through dairy waste valorization. The results showed that the immobilization on Pissa Pafos favored the viability of *L. casei* cells. In parallel, the antimicrobial properties of the resin resulted in an extended shelf life of whey beverages compared to pasteurized cheese whey, indicating the high commercialization potential of the products. Likewise, the present study highlights an alternative cheese whey valorization process, contributing toward the sustainable transition to a bioeconomy era.

## Figures and Tables

**Table 1:** Determination of pH, total acidity, glucose, galactose, and lactose content of whey beverages during 30 days of refrigerated (4 °C) storage.

Whey Beverage	Storage Time (Days)	pH	Total Acidity ^1^	Glucose ^2^	Galactose ^2^	Lactose ^2^
**C**	1	3.92 ± 0.02 ^a^	0.6 ± 0.04 ^a^	0.30 ± 0.01 ^a^	0.18 ± 0.01 ^a^	1.82 ± 0.01 ^a^
	15	3.93 ± 0.02 ^a^	0.6 ± 0.04 ^a^	0.27 ± 0.02 ^a^	0.18 ± 0.01 ^a^	1.80 ± 0.01 ^a^
	30	4.02 ± 0.02 ^b^	0.5 ± 0.04 ^b^	0.22 ± 0.01 ^b^	0.11 ± 0.01 ^b^	1.80 ± 0.01 ^a^
**A1**	1	3.90 ± 0.02 ^a^	0.7 ± 0.04 ^a^	0.16 ± 0.01 ^a^	0.20 ± 0.02 ^a^	1.80 ± 0.01 ^a^
	15	3.69 ± 0.02 ^b^	0.7 ± 0.03 ^a^	0.13 ± 0.01 ^b^	0.21 ± 0.02 ^a^	1.79 ± 0.01 ^a^
	30	3.67 ± 0.02 ^b^	0.7 ± 0.03 ^a^	0.11 ± 0.01 ^b^	0.23 ± 0.02 ^a^	1.70 ± 0.01 ^b^
**A2**	1	3.90 ± 0.02 ^a^	0.7 ± 0.04 ^a^	0.17 ± 0.01 ^a^	0.20 ± 0.02 ^a^	1.78 ± 0.01 ^a^
	15	3.71 ± 0.02 ^b^	0.7 ± 0.03 ^a^	0.13 ± 0.01 ^b^	0.22 ± 0.02 ^a^	1.70 ± 0.01 ^b^
	30	3.64 ± 0.02 ^c^	0.7 ± 0.04 ^a^	0.10 ± 0.02 ^b^	0.21 ± 0.02 ^a^	1.65 ± 0.02 ^c^
**A3**	1	3.90 ± 0.02 ^a^	0.7 ± 0.03 ^a^	0.17 ± 0.01 ^a^	0.25 ± 0.02 ^a^	1.81 ± 0.01 ^a^
	15	3.71 ± 0.02 ^b^	0.7 ± 0.04 ^a^	0.14 ± 0.02 ^b^	0.23 ± 0.02 ^a^	1.80 ± 0.01 ^a^
	30	3.71 ± 0.02 ^b^	0.7 ± 0.04 ^a^	0.13 ± 0.05 ^b^	0.24 ± 0.02 ^a^	1.77 ± 0.01 ^b^
**B1**	1	3.93 ± 0.02 ^a^	0.6 ± 0.03 ^a^	0.33 ± 0.02 ^a^	0.18 ± 0.01 ^a^	1.83 ± 0.01 ^a^
	15	3.91 ± 0.02 ^a^	0.6 ± 0.04 ^a^	0.31 ± 0.02 ^a^	0.18 ± 0.02 ^a^	1.83 ± 0.01 ^a^
	30	3.88 ± 0.02 ^a^	0.6 ± 0.04 ^a^	0.32 ± 0.02 ^a^	0.15 ± 0.03 ^a^	1.83 ± 0.01 ^a^
**B2**	1	3.93 ± 0.02 ^a^	0.6 ± 0.04 ^a^	0.31 ± 0.02 ^a^	0.17 ± 0.01 ^a^	1.80 ± 0.01 ^a^
	15	3.91 ± 0.03 ^a^	0.6 ± 0.04 ^a^	0.32 ± 0.02 ^a^	0.15 ± 0.03 ^a^	1.80 ± 0.01 ^a^
	30	3.90 ± 0.02 ^a^	0.6 ± 0.04 ^a^	0.33 ± 0.02 ^a^	0.15 ± 0.02 ^a^	1.81 ± 0.02 ^a^
**B3**	1	3.94 ± 0.02 ^a^	0.6 ± 0.04 ^a^	0.33 ± 0.02 ^a^	0.18 ± 0.01 ^a^	1.83 ± 0.01 ^a^
	15	3.92 ± 0.02 ^a^	0.6 ± 0.04 ^a^	0.34 ± 0.02 ^a^	0.17 ± 0.01 ^a^	1.82 ± 0.01 ^a^
	30	3.91 ± 0.02 ^a^	0.6 ± 0.04 ^a^	0.32 ± 0.02 ^a^	0.18 ± 0.02 ^a^	1.82 ± 0.01 ^a^

^1^ Expressed as % lactic acid; ^2^ Expressed as g per 100 mL whey beverage; Different letters (a, b, c) indicate significant differences (*p* < 0.05) among different storage days for each whey beverage.

**Table 2:** Volatile compounds identified by solid-phase microextraction gas chromatography/mass spectrometry (SPME/GC-MS) in whey beverages (A2: 0.6 g of the immobilized biocatalyst per 100 mL of whey; B2: 0.6 g of *Pistacia terebinthus* resin per 100 mL of whey; C: control sample) after 30 days of storage (all values presented as percentage).

Compounds	ID *	C	A2	B2
***Esters***				
ethyl acetate	RT, KI, MS	1.4	0.7	1.3
ethyl butanoate	RT, KI, MS	2.7	1.3	2.1
propyl butanoate	MS	Nd	0.1	0.2
ethyl pentanoate	MS	Nd	0.1	0.1
butyl butanoate	MS	1.3	0.8	1.2
ethyl hexanoate	RT, KI, MS	2.5	1.5	1.9
ethyl heptanoate	RT, MS	Nd	Tr	0.2
ethyl octanoate	RT, KI, MS	3.4	1.7	3.1
ethyl nonanoate	RT, MS	Nd	0.1	0.1
ethyl decanoate	RT, KI, MS	1.2	0.8	0.8
ethyl dodecanoate	KI, MS	0.8	0.5	0.5
ethyl tetradecanoate	KI, MS	Nd	0.1	Nd
Sum esters/Sum total compounds		13.2	7.7	11.5
***Organic acids***				
boutanoic acid	RT, MS	4.1	2.7	3.3
hexanoic acid	RT, MS	11.6	11.6	10.6
2-methyl-butanoic acid	KI, MS	Nd	0.6	Nd
3-methyl-butanoic acid	KI, MS	Nd	0.9	0.1
octanoic acid	RT, KI, MS	13.5	16.2	10.3
nonanoic acid	RT, KI, MS	2.8	1.5	2.1
decanoic acid	RT, KI, MS	8.7	14.5	7.4
Sum acids/Sum total compounds		40.6	48.1	33.7
***Alcohols***				
2-pentanol		Nd	0.3	Nd
2-methyl-1-butanol	RT, MS	Nd	0.3	Nd
3-methyl-1-butanol	RT, MS	1.6	Nd	Nd
2-heptanol	RT, MS	Nd	0.2	Nd
1-hexanol	RT, KI, MS	9.6	2.4	6.6
1-octen-3-ol	RT, KI, MS	2.5	1.5	3.1
1-heptanol	RT, KI, MS	1.2	1.4	Nd
2-ethyl-1-hexanol	RT, KI, MS	1.8	1.3	1.7
1-octanol	RT, KI, MS	1.0	1.0	0.2
phenylethyl alcohol	RT, KI, MS	0.9	2.2	2.4
Sum alcohols/Sum total compounds		18.7	10.5	14.0
***Aldehydes***				
3-methyl butanal	RT, MS	1.6	1.0	2.3
hexanal	RT, KI, MS	6.0	2.4	3.9
heptanal	RT, KI, MS	2.9	1.7	3.4
octanal	RT, KI, MS	1.1	0.9	Nd
2-pentenal	RT, MS	Nd	0.7	Nd
2-heptenal	RT, KI, MS	Nd	Nd	0.1
2-octenal	RT, MS	1.3	0.8	2.2
nonanal	RT, MS	Nd	0.2	Nd
decanal	RT, KI, MS	1.2	0.5	1.1
benzaldehyde	RT, KI, MS	1.1	0.7	1.3
Sum aldehydes/Sum total compounds		15.4	8.9	14.4
***Ketones***				
2-butanone	RT, KI, MS	3.7	4.6	3.1
2-pentanone	RT, MS	2.7	3.1	3.3
2-heptanone	RT, MS	1.9	3.3	2.5
2-nonanone	RT, MS	2.8	3.9	1.9
2,3-butanedione	RT, KI, MS	1.0	3.0	Nd
Sum ketones/Sum total compounds		12.1	17.9	10.7
***Monoterpenes***				
a-pinene	KI, MS	Tr	5.3	11.6
camphere	KI, MS	Nd	0.1	0.2
β-pinene	KI, MS	Nd	0.3	0.7
3-carene	KI, MS	Nd	Tr	0.1
β-myrcene	KI, MS	Nd	Tr	0.1
2-carene	KI, MS	Nd	Nd	0.0
D-limonene	KI, MS	Tr	0.1	0.3
Beta-phellandrene	KI, MS	Nd	Tr	0.0
o-cymene	KI, MS	Nd	0.1	0.2
Sum monoterpenes/Sum total		Tr	5.9	13.2
***Oxygenated Monoterpenes***				
eucalyptol	KI, MS	Nd	0.1	0.1
terpinolene	KI, MS	Nd	0.2	0.5
linalool	KI, MS	Nd	0.1	0.2
4-terpineol	KI, MS	Nd	0.1	0.2
pinocarveol	KI, MS	Nd	0.1	0.2
verbenol	KI, MS	Nd	Tr	0.1
α-terpineol	KI, MS	Nd	0.3	0.8
melilotal	KI, MS	Nd	Tr	0.1
myrtenol	KI, MS	Nd	Tr	0.1
p-cymene-8-ol	KI, MS	Nd	0.1	0.2
Sum oxyg. monoterpenes/Sum total		Tr	1.0	2.5

* ID: Method of identification, KI: tentative identification by Kovats retention index in accordance with literature [22,23,25,37,67,68,69,70,71], RT: Positive identification by retention times that agrees with authentic compounds and by the mass spectra of authentic compounds generated in the laboratory, MS: tentative identification by mass spectra obtained from NIST107, NIST21, and SZTERP libraries, Nd: not detected, Tr: Traces (<0.1%).
